# A Corrector Corrected

**Published:** 1890-05

**Authors:** 


A Corrector Corrected; *The Boston Evening Transcript*, March 22d.

This is the title to a very clever letter to the editor of the *Transcript*, and signed "A Hahnemannian Homœopathist." It is evidently from the pen of our well-known friend Dr. Samuel A. Kimball.

It will be remembered that in the March number, at page 141, we called attention to an article by Mark Twain in *Harper's*, upon Homœopathy. The same article is again considered in the present number at page 203.

It seems that some "corrector" of allopathic partialities took Mark Twain to task on account of his preferences for Homœopathy. He brought up the old accusation that Hahnemann attributed seven-eighths of the diseases of humanity to *the itch*.

The letter here referred to is a caustic and conclusive answer to the exceptions of "Corrector." W. M. J.

